# An Investigation of the Coherence of Oral Narratives: Associations With Mental Health, Social Support and the Coherence of Written Narratives

**DOI:** 10.3389/fpsyg.2020.602725

**Published:** 2021-01-13

**Authors:** Lauranne Vanaken, Patricia Bijttebier, Dirk Hermans

**Affiliations:** ^1^Centre for the Psychology of Learning and Experimental Psychopathology, Faculty of Psychology and Educational Sciences, KU Leuven, Leuven, Belgium; ^2^School Psychology and Development in Context, Faculty of Psychology and Educational Sciences, KU Leuven, Leuven, Belgium

**Keywords:** autobiographical memory, social function of autobiographical memory, narrative coherence, oral modality, narrative style

## Abstract

**Research Questions:**

In a first research question, we examined whether the relations that are generally observed between the coherence of written autobiographical narratives and outcomes of mental health and social support, can be replicated for the coherence of oral narratives. Second, we studied whether the coherence of oral narratives is related to the coherence of written narratives.

**Methods:**

Pearson correlations and *t*-tests were calculated on data of two separate studies to examine the research questions.

**Results:**

First, only thematic coherence of oral narratives was significantly, although moderately, negatively associated to symptoms of depression, anxiety and negative social interactions. Second, the coherence of oral narratives was higher than the coherence of written narratives. Only the thematic coherence of oral narratives was positively associated with thematic and total coherence of written narratives. Furthermore, correlations between written and oral narratives were stronger for negative narratives as compared to positive narratives.

**Discussion:**

The ability to elaborate emotionally and make meaning out of important life events in oral narratives is, to a certain extent, related to better mental health and more social support. Furthermore, thematic coherence may be a relatively stable feature of individuals’ narrative styles that is reflected in narratives of different modalities. Nonetheless, these topics need to be further researched to overcome present limitations.

## Introduction

### Associations Between the Coherence of Oral Narratives, Mental Health and Social Support

On a daily basis we talk about the highs and lows of our lives to the people around us (Rimé et al., 1992, 1998; Rimé, 2009). These highs and lows, or significant personal experiences are what constitute the autobiographical memory and it is no coincidence that they are socially shared frequently (Tulving, 1972; Fivush, 2011). Notably through collaborative remembering, the social function of autobiographical memory can be fulfilled (Bruce, 1989; Pillemer, 1992; Bluck and Alea, 2002; Bluck, 2003; Bluck et al., 2005; Meade et al., 2018). This social function concerns the development, maintenance and enhancement of social bonds through the sharing of autobiographical narratives and has shown to be of important value for our psychological well-being (Alea and Bluck, 2003, 2007). As a matter of fact, by sharing autobiographical memories, our social network and specifically the support we receive from that network can be enhanced (Coyne, 1976; Barry et al., 2019; Vanaken and Hermans, 2020a; Vanaken et al., 2020). Social support has in turn shown to be pivotal with regards to maintaining and improving our mental health (Cohen and Wills, 1985; Ozbay et al., 2007; Sippel et al., 2015; Harandi et al., 2017).

However, the extent to which the social function is successfully fulfilled by sharing autobiographical memories and consequent support is received, is in part dependent on the distinct ways in which these memories are narrated (e.g., Alea and Bluck, 2003; Barry et al., 2019; Vanaken and Hermans, 2020a; Vanaken et al., 2020). One of the aspects of narratives that might be of importance here is coherence (Reese et al., 2011; Vanaken and Hermans, 2020a; Vanaken et al., 2020). Whilst coherence has been defined and used transdisciplinary in slightly differing ways (Baerger and McAdams, 1999; Habermas and Bluck, 2000; Lysaker et al., 2002; Reese et al., 2011; Adler et al., 2018), it is generally agreed upon that it is a cornerstone of communication (Labov, 1970). Broadly defined, narrative coherence is the extent to which a narrative “makes sense to a naïve listener–not just in terms of understanding when, where, and what event took place–but also with respect to understanding the meaning of that event to the narrator” (Reese et al., 2011, p. 425).

As coherence has been repeatedly designated as one of the necessary conditions of high-quality narration (McAdams, 2006; Adler et al., 2007; Reese et al., 2011), it has been receiving increased research interest ever since (e.g., Baerger and McAdams, 1999; Habermas and Bluck, 2000; Reese et al., 2011, 2014, 2017; Habermas and Reese, 2015; Waters and Fivush, 2015). Specifically, narrative coherence has been investigated in relation to various outcomes of social support and mental health, however yielding mixed results overall.

For instance, Burnell et al. (2010) observed that veterans who were more coherent in their oral narration, experienced communication with friends and family as more positive, whereas incoherent veterans reported that their communication remained unsatisfactory. Similarly, Waters and Fivush (2015) found that narrative coherence was positively related to both psychological well-being and positive social relationships, as measured by perceived social support, social well-being, and feelings of generativity. In a recent study of Vanaken et al. (unpublished), written narrative coherence was negatively associated with negative social interactions, but no relations were found with positive social interactions.

With regards to mental health, multiple outcomes of psychological well-being and psychopathology have been investigated over the years. Baerger and McAdams (1999) observed that adults who orally narrated stories from their lives more coherently reported lower levels of depression and higher life satisfaction. In studies from Waters and Fivush (2015), as well as from Reese et al. (2011), positive relations between written narrative coherence and psychological well-being were found. However, in a later study of Reese et al. (2017), higher written narrative coherence related to lower levels of well-being for older adolescents, whereas younger adolescents with higher coherence reported poorer well-being instead. Moreover, Vanderveren et al. (2019) and Mitchell et al. (2020) observed negative associations between the written coherence of narratives and symptoms of depression, but not anxiety. Furthermore, low levels of coherence in narratives are suggested to be associated with of psychiatric problems like borderline personality disorder and schizophrenia (e.g., Hermans, 2006; Lysaker and Lysaker, 2006; Raffard et al., 2010; Adler, 2012). Nonetheless, Adler et al. (2007, 2008) found the coherence of psychotherapy narratives to be associated with ego development, but not with psychological well-being. Moreover, symptoms of trauma or PTSD have been shown to be associated with lower levels of coherence in written narratives (Brewin et al., 1996; Brewin, 2001; Tuval-Mashiach et al., 2004), however also oftentimes with contradicting results (Rubin et al., 2008, 2016; Rubin, 2011; Waters et al., 2013). In sum, different results can be observed dependent on the age and gender of the participants (e.g., McLean et al., 2010; Chen et al., 2012; Reese et al., 2017), on the outcome variable under investigation (e.g., Vanderveren et al., 2019), or on the specific subdimension of coherence (e.g., Mitchell et al., 2020).

Possibly, one source of inconsistencies in the results might arise from these differences in measurement methods. For instance, in addition to differences in research samples, conceptualizations and coding schemes for coherence, also narratives of different event types and in different modalities have been used in the investigation of coherence (e.g., McLean et al., 2017). To date, not much is known about if and how these methodological differences can impact the relation between coherence and outcomes of mental health or social support.

Particularly with regards to social support, it would be crucial to compare relations to coherence of oral versus written narratives. To our knowledge, there has been no research that directly compares the relations between coherence in different modalities and outcomes of mental health or social support, within the same person. There is some work that does compare modalities of remembering in relation to well-being, but none of these include the fundamental characteristic of coherence. For instance, Lyubomirsky et al. (2006) found that individuals who processed a negative life event through writing or talking reported improved well-being in comparison to those who thought about it. The reverse associations were observed when participants focused on positive life events.

Therefore, in our *first* research question, we want to examine if the associations to mental health and social support that have sometimes been observed for the coherence of written narratives, can be observed for the coherence of oral narratives. We hypothesize that coherence of oral narratives will be positively associated to psychological well-being and positive social interactions, and negatively to symptoms of depression, anxiety, stress and to negative social interactions. This hypothesis is based on a critical evaluation of the existing literature, which yields mixed results but is nonetheless still to a larger extent in support of the idea that narrative coherence might be beneficial for social and psychological adjustment, at least in older adolescents and adults (e.g., Baerger and McAdams, 1999; Burnell et al., 2010; Reese et al., 2011; Chen et al., 2012; Waters and Fivush, 2015; Adler et al., 2016; Vanderveren et al., 2019; Vanaken et al., unpublished).

### Associations Between the Coherence of Oral and Written Narratives

Furthermore, whilst narrative findings are often transferred across studies with different methodologies, not much is known about if and how oral and written narratives can compare in terms of narrative coherence. In day-to-day life, the use of oral narratives is more common in real-life social interactions, whereas written narratives are more likely to be used via social media (Rimé, 2009; Habermas, 2019). In the scientific analysis of narratives, written narratives are most often used, since they are a more economic, feasible and practical way to study autobiographical reminiscing behavior (e.g., Pennebaker et al., 1997). Nonetheless, the assessment of coherence in oral modality is quite common in some domains (e.g., Life Story Interview: Baerger and McAdams, 1999). However, this method of using life stories is not really convenient to study the application to day-to-day social interactions since these narratives are often rather long. Therefore, it would be interesting to investigate how oral and written narratives of shorter, nuclear life episodes compare.

Since the 70’s, narratives of different modalities have been compared in terms of various structural variables. Some research suggests that written autobiographical narratives are more reflected upon than oral ones, which could yield the former more complete, complex and coherent than the latter (Tannen, 1982; Eaton, 2005; Drijbooms et al., 2017). However, other research indicates little to no differences between oral and written narratives (Chafe and Tannen, 1987; Balon and Rimé, 2016). Some studies also find mixed evidence depending on the indicator used. For instance, written narratives from university students contained more general evaluative sentences but were shorter in length and contained fewer emotional evaluations (Özyildirim, 2009). Sometimes, it is suggested that oral narratives can be more complete or coherent than written ones (Tannen, 1982). Possibly, this might be the case because “oral narratives may also have some degree of reflected-upon, planned character, such as planned public speeches, cultural stories, like fairy tales, or frequently retold anecdotes” (Habermas, 2019, p. 23). In sum, evidence about differences and similarities in structural variables, amongst which coherence, between written and oral narratives is not conclusive yet.

When comparing different modalities of narratives in terms of coherence, it can be useful to take into account how narrative coherence is conceptualized. Narrative coherence is thought to partly reflect a relatively stable personal style (i.e., a certain degree of intra-individual stability), once developed (e.g., Reese et al., 2011; Waters et al., 2019; Pasupathi et al., 2020). For instance, different measurements of coherence are observed to be moderately correlated within individuals over time and event-type (McLean et al., 2017; Waters et al., 2019; Vanaken et al., unpublished). This is line with the idea that coherence is suggested to be partly determined by event-specific factors (e.g., type of event: Banks and Salmon, 2013; emotional intensity: Rubin et al., 2011) and narration-specific factors (e.g., context of sharing: Bavelas et al., 2000; Pasupathi, 2001), yielding a certain extent of intra-individual variability (Fivush et al., 2017; McLean et al., 2017; Waters et al., 2019; Pasupathi et al., 2020). For instance, both inherent differences between writing and speaking (e.g., Grysman, 2015), as well as social-contextual differences that are associated to different modalities (Bavelas et al., 2000; Pasupathi, 2001; Alea and Bluck, 2003) could impact the narrative. However, not much is known about the extent to which the persons’ narrative style can be apparent across narratives of different modalities, or to what extent certain variables (like modality, event type, or timing) can differentially impact the coherence of the narration.

In sum, research on differences in structural variables, like coherence, between oral and written narratives is still inconclusive. Furthermore, we do not know much yet about the impact of the modality on the intra-individual stability of characteristics of narratives. Hence, our *second* research question involves whether coherence of oral narratives is related to coherence of written narratives. We hypothesize to observe a moderate association between oral and written narrative coherence, based on results of previous studies suggesting the combinatory impact of both stable and variable elements in the construction of coherent narratives (Fivush et al., 2017; McLean et al., 2017; Waters et al., 2019; Pasupathi et al., 2020).

### Present Study

The data in this manuscript stem from two separate studies, that were executed with 4 months in between, in the same sample of participants (Vanaken and Hermans, 2020b; Vanaken et al., unpublished) Whilst the two studies were set up independently and with separate pre-registered research aims, the combination of both allowed us to test the aforementioned hypotheses. One study measured coherence in written narratives (Vanaken et al., unpublished)^1^, and one measured coherence in oral narratives in the same sample of participants 4 months later (Vanaken and Hermans, 2020b)^2^. The comparison of results from both studies, allowed us to investigate if the coherence of oral narratives is related to mental health and social support, as well as to the coherence of written narratives.

## Materials and Methods

### Participants

The sample consisted of 68 individuals, of whom 10 were men (14.7%) and 58 were women (85.3%), ranging in age between 17 and 20, *M* = 18.31, *SD* = 0.68. All participants were first-year psychology students.

### Measurements

#### Questionnaires

##### Psychological well-being

Psychological well-being was measured using the Flourishing Scale (FS) (Diener et al., 2009; Dutch translation: van Egmond and Hanke, n.d.). This instrument consists of eight items to assess the respondent’s self-perceived psychosocial prosperity and has shown to be related to the full version of the psychological well-being scales that Ryff (1989) developed. It is a brief measurement that provides a single score of psychological well-being, which has shown to be highly internal reliable, Cronbach’s α = 0.86, and highly temporally stable, *r* = 0.71 (Diener et al., 2009). Example items are for instance: “I lead a purposeful and meaningful life,” or “I am optimistic about my future.”

##### Depression, anxiety, stress

To assess internalizing symptoms of psychopathology, we used the Dutch version of the Depression (D), Anxiety (A), and Stress (S) Scales (DASS-21: Lovibond and Lovibond, 1995; Dutch translation: De Beurs et al., 2001). This instrument assesses self-reported symptoms of depression, anxiety and stress, and has shown to be internally consistent, 0.85 ≤ Cronbach’s α ≤ 0.91, test–retest reliable, 0.74 ≤ *r* ≤ 0.85, and valid in a Dutch sample of first year university students, *N* = 289, which is similar to our sample (De Beurs et al., 2001). Example items include “I couldn’t seem to experience any positive feeling at all” (D), I felt scared without any good reason (A), and “I found it difficult to relax” (S).

##### Social interactions

We assessed social interactions with the Social Support List – Interactions (SSL-I) and – Negative Interactions (SSL-N), which have also proven to have good construct validity, high internal reliability, SSL-I:0.90 ≤ Cronbach’s α ≤ 0.93; SSL-N:0.69 ≤ Cronbach’s α ≤ 0.81, and test–retest stability, SSL-I: *r* = 0.77; SSL-N: *r* = 0.56 (Van Sonderen, 2012). Research has demonstrated that negative interactions (e.g., giving one disapproving comments, treating one unfairly), are not at the other end of the spectrum of positive interactions. They are regarded as an independent aspect of interpersonal functioning and are related to psychological non-well-being (Van Sonderen and Ormel, 1991). Example items for the positive interactions are: “People confide in you,” “People are affectionate toward you,” and “People ask you to join in.” Example items for the negative interactions are: “People make disapproving remarks toward you,” “People treat you unjustly,” and “People react coolly.”

#### Narrative Coherence

##### Written modality

In the narrative coherence writing tasks, participants were instructed to write for a minimum time of 10 min about an autobiographical high and low point in their life (in counterbalanced order across participants). Participants were encouraged to take this task very seriously, and to really take their time to think and write about impactful events, as no maximum time to do so was given. The specific instructions were as follows: “I would like for you to write about your most positive/negative experience of your life. This should be an extremely emotional event that has affected you and your life. You may include the facts of the event, as well as your deepest thoughts and feelings. All of your writing will be kept confidential. Do not worry about spelling, sentence structure, or grammar. There is no time limit on your writing; you may write about this event for as long as needed.”

##### Oral modality

Participants were asked to narrate out loud about a turning point memory in their life for a minimum time of 5 min. The following instructions were presented on the computer screen during the entire narration process: “I would like you to think back over your life and identify an event that has changed your life or the kind of person you are. It could be something from any area of your life–your relationships with other people, your work and school, your outside interests, and so forth. Please identify a particular episode in your life story that you now see as a turning point in what your life is like or what you are like as a person. Please describe what happened, when it happened, who was involved, what you were thinking and feeling, why this experience is significant, and how it changed your life or you as a person.”

##### Rating

The oral memories were first transcribed into written narratives. Afterward, all narratives were rated manually according to the Narrative Coherence Coding Scheme (NaCCS) (Reese et al., 2011). Using this coding scheme, each narrative was assigned a total score from 0 to 9, consisting of the sum of the scores on the three dimensions that the scheme entails, namely context (0–3), chronology (0–3), and theme (0–3). Context was assessed based on whether time and place of the events were described and specified. Chronology was scored according to the logical and chronological order in which events were outlined, as well as according to the use of time indicator words to enhance narrative structure. Theme was assessed by investigating whether the narrator did not merely describe facts, but also created meaning, linked events to other autobiographical events, came to a resolution or reached closure. Total narrative coherence (0–9) was calculated by summing the scores for the individual dimensions.

All written narratives were rated independently by three trained coders, upon which a consensus system was used to determine final scores (i.e., all narratives were discussed together and given a final score based on the score that the majority of raters had given or agreed to give in case of initially different scorings). For the oral narratives, we used the standard approach in the domain to code narratives. Namely, we established good inter-rater reliability on 10% of the data, as was evident from a high interclass correlations (ICC) for all dimensions, *ICC*_*context*_ = 0.94; *ICC*_*chronology*_ = 0.88; *ICC*_*theme*_ = 0.90. Afterward, all other oral data were rated by the first author.

### Procedure

The written measurement of coherence was conducted as part of a first-year psychology course “Methods of Research,” in which students are given the opportunity to contribute to science and gain experience in research practices. Participants were collectively informed about the aims and procedure of the study. Then, after signing agreement to the informed consent, they could individually and online complete the questionnaires and the writing tasks, which involved writing about a high point memory and a low point memory. Instructions were given to do this in a quiet space with no distractions and to respond to all questions as honestly possible. After participation, students were given further information about the specific aim of the study, contact details of the research team, professional help instances, a course credit and they were thanked for their effort and time. This study was conducted in accordance with ethical guidelines and approved by the Social and Societal Ethics Committee of the KU Leuven (G-2018 01 1067).

The oral measurement took place 4 months after the written measurement, and was part of an experimental study, for which individuals who participated in the written measurement were invited via e-mail. In the experiment, participants were individually invited to the lab, where they were informed about the aims and procedure of the study. Then, after signing agreement to the informed consent, they could individually fill out the DASS-depression items on the computer. No other questionnaires were administered during this part of the study. Afterward, they were asked to narrate out loud about a turning point memory. Important to note here is that half of the participants in this study told the narrative in the presence of an experimenter with an evaluative attitude and in front of a camera, which was part of the experimental manipulation in that study. The other half of the participants presented their narrative into a microphone whilst being alone in the cubicle. In fact, all oral narratives were audio recorded and later transcribed to be rated on coherence. After participation, students were given further information about the specific aim of the study, contact details of the research team, professional help instances, a course credit and they were thanked for their effort and time. This study was conducted in accordance with ethical guidelines and approved by the Social and Societal Ethics Committee of the KU Leuven (G-2018 10 1376).

### Analyses

Results were analyzed using the coherence scores on the individual dimensions (context, chronology, theme), as well as total narrative coherence scores. For the written narratives, the mean of the dimensional and total scores on high point and low point narratives were used. For the oral narratives, the dimensional and total scores on the turning point narrative were used. Pearson’s correlations were calculated using SPSS version 26. An alpha level of 0.05 was set for all analyses. Anonymized data have been made publicly available at the Open Science Framework and can be accessed^3^.

## Results

### Descriptives

Descriptive statistics were calculated for the dimensions of coherence as well as the total coherence score in written and oral narratives. The ranges, means and standard deviations are presented in Table 1. Coherence scores were generally higher for oral narratives than for written narratives. This difference was significant for contextual, *t*(67) = 3.49, *p* = 0.001, thematic, *t*(67) = 5.70, *p* < 0.001, and total narrative coherence, *t*(67) = 4.17, *p* < 0.001, but not for chronological coherence, *t*(67) = 1.15, *p* = 0.25.

**TABLE 1:** Descriptive statistics for written and oral narrative coherence.

	**Oral**				**Written**			
	**Min**	**Max**	**M**	**SD**	**Min**	**Max**	**M**	**SD**
CON	1	3	2.16	0.99	0	3	1.63	0.81
CHR	0	3	2.15	0.97	0	3	1.99	0.87
THE	0	3	2.62	0.71	0	3	2.00	0.82
TOT	2	9	6.93	1.98	0	9	5.61	2.15

*CON, contextual coherence; CHR, chronological coherence; THE, thematic coherence; TOT, total coherence.*

### Associations Between the Coherence of Oral Narratives, Mental Health and Social Support

We calculated Pearson correlations between the coherence of oral narratives and outcomes of mental health and social support, that were assessed either during the written measurement 4 months earlier or at the time of the oral measurement itself. Note that only depressive symptoms were measured at both times, whereas all other measures were collected during the written measurement.

In Table 2, the relations between the coherence of oral narratives and the investigated outcomes are presented. At the same moment in time, only thematic coherence of oral narratives was significantly negatively related to symptoms of depression. In other words, the more individuals were able to make meaning out of a turning point event in their oral narratives, the less depressed they felt concurrently. Furthermore, also only thematic coherence of oral narratives was significantly negatively associated with symptoms of anxiety and with negative social interactions, both of the latter measured 4 months earlier in time. When people experienced fewer anxious feelings and fewer negative social interactions, they were better able to create a thematically meaningful narrative 4 months later. Relations to symptoms of stress and positive social interactions were not significant.

**TABLE 2:** Correlations between oral narrative coherence, mental health and social support.

	**Oral**					**Written**			
	**CON**	**CHR**	**THE**	**TOT**		**CON**	**CHR**	**THE**	**TOT**
DEPR conc	–0.09	–0.20	−0.27*	–0.24	DEPR conc	−0.12*	−0.17**	−0.11*	−0.17**
DEPR -4 m	0.08	0.00	–0.08	0.01	DEPR +4 m	0.01	–0.03	–0.08	–0.04
PWB -4 m	–0.13	–0.01	0.04	–0.05	PWB conc	0.12*	0.11*	0.15**	0.16**
ANX -4 m	–0.03	0.00	−0.29*	–0.12	ANX conc	−0.14**	−0.13*	–0.04	−0.13*
STR -4 m	0.00	0.05	–0.03	0.02	STR conc	–0.06	–0.03	0.08	–0.01
SS_P -4 m	–0.14	0.08	–0.02	–0.04	SS_P conc	0.09	0.05	0.05	0.08
SS_N -4 m	0.13	–0.09	−0.38**	–0.12	SS_N conc	−0.12*	−0.14**	−0.11*	−0.15**

CON, contextual coherence; CHR, chronological coherence; THE, thematic coherence; TOT, total coherence; DEPR, depressive symptoms; PWB, psychological well-being; ANX, symptoms of anxiety; STR, symptoms of stress; SS_P, social support: positive social interactions; SS_N, social support: negative social interactions; conc, measured concurrently; −4 m, measured 4 months earlier (during the written narrative study), +4 m, measured 4 months later (during the oral narrative study). Bold values indicate the significant. * *p* < 0.05. ** *p* < 0.0125 (Bonferroni adjusted).

In Table 2 as well, the relations between written narrative coherence and the investigated outcomes are shown, as a base for comparison. Comparing the data of oral narratives to the data of written narratives, we can observe more extended pattern of relations between written narrative coherence and outcomes of mental health and social support, as compared to oral narrative coherence. Specifically, there were significantly positive relations between all forms of written coherence and the concurrent measure of psychological well-being, and significant negative relations between all forms of written coherence and concurrent measures of depressive symptoms, symptoms of anxiety (except for thematic coherence), symptoms of stress and negative social interactions. The fact that these outcomes were measured concurrently for the written narratives might have likely played a large role in the more extended pattern of results. We will elaborate on this in the discussion.

Bonferroni corrections were applied since correlations were calculated for four measures of coherence (context, chronology, theme, total). Data can thus be compared to an adjusted alpha level of 0.0125. For oral narratives, only the negative association between thematic coherence and negative social interactions, remained significant. For written narratives, multiple correlations remained significant when compared to the adjusted alpha (see ^∗∗^ in Table 2). Note that Bonferroni corrections might be too conservative, since the aims of the study were explorative in nature and the study was carried out to have preliminary findings inspire future preregistered research.

### Associations Between the Coherence of Oral and Written Narratives

Pearson correlations were calculated to investigate the relations between oral and written modalities of narrative coherence. Results are presented in Table 3. We observed that only the thematic coherence of oral narratives related positively to the coherence of written narratives. In particular, thematic coherence of oral narratives was significantly associated to the contextual, thematic and total coherence of written narratives.

**TABLE 3:** Relations between oral and written narrative coherence.

		**O_CON**	**O_CHR**	**O_THE**	**O_TOT**
W_CON	*r*	0.01	0.09	0.25*	0.14
W_CHR	*r*	0.06	0.21	0.23	0.22
W_THE	*r*	0.01	0.12	0.33**	0.18
W_TOT	*r*	0.03	0.16	0.32**	0.21

W, written narratives; O, oral narratives; CON, contextual coherence; CHR, chronological coherence; THE, thematic coherence; TOT, total coherence. Bold values indicate the significant. * *p* < 0.05. ** *p* < 0.0125 (Bonferroni adjusted).

A paired-samples *t*-test pointed out that the negative and positive written narratives were significantly different in terms of coherence, *t*(67) = −2.37, *p* = 0.21. More specifically, negative written narratives were generally more coherent than written positive narratives, *M*_*pos*_ = 5.32, *SD*_*pos*_ = 2.43, *M*_*neg*_ = 5.90, *SD*_*neg*_ = 2.32. Therefore, we investigated if the coherence of oral narratives was similarly related to the coherence in positive written versus negative written narratives. Table 4 shows a similar pattern of results for positive written and negative written narratives. Correlations to thematic coherence of oral narratives were found for thematic and total coherence, both in positive written and negative written narratives. Nonetheless, the associations were relatively stronger for negative narratives, in comparison to positive narratives.

**TABLE 4:** Relations between oral and written narrative coherence, separately for negative and positive written narratives.

	**O_CON**	**O_CHR**	**O_THE**	**O_TOT**
W_CON_NEG	−0.10	0.05	0.21	0.05
W_CHR_NEG	0.08	0.20	0.22	0.21
W_THE_NEG	0.06	0.13	0.35**	0.22
W_TOT_NEG	0.01	0.15	0.32**	0.19
W_CON_POS	0.12	0.09	0.18	0.17
W_CHR_POS	0.04	0.18	0.20	0.18
W_THE_POS	−0.04	0.09	0.25*	0.11
W_TOT_POS	0.05	0.14	0.25*	0.19

W, written narratives; O, oral narratives; CON, contextual coherence; CHR, chronological coherence; THE, thematic coherence; TOT, total coherence. Bold values indicate the significant. * *p* < 0.05. ** *p* < 0.0125 (Bonferroni adjusted).

After applying Bonferroni corrections for testing coherence multiple times, α = 0.0125, only the associations of oral thematic coherence with written thematic and written total coherence remained significant, both for total narrative coherence scores, as well as for the negative narratives separately.

## Discussion

In the present study, the *first* question concerned the associations of the coherence of oral narratives with mental health and social support. Results indicated that relations that are generally observed for written narrative coherence were also evident for oral narrative coherence, but for only the thematic dimension. Only thematic coherence in oral narratives was negatively associated with concurrent depressive symptoms, and retrospectively negatively associated with anxiety symptoms and perceived negative social interactions. After Bonferroni corrections, only the correlation between thematic coherence of oral narratives and earlier reported negative social interactions remained significant. For written narratives, a more extended pattern of associations was observed, with more measures of coherence being related to mental health and social support.

Important to notice however, is that this more extended pattern of results for written narratives is likely dependent on the fact that outcome measures were collected concurrently for the written narratives, whereas 4 months before the oral narratives were collected. Our results indicate that coherence might be particularly relevant for concurrent adjustment, but that associations decrease in strength over time. This is also confirmed by our findings that the coherence of both oral and written narratives was significantly negatively associated with the concurrent measure of depressive symptoms, but not with the depressive symptoms measured either 4 months earlier or later.

Overall, significant results were not observed for every measure of coherence and every outcome. Hence, these findings are still to be interpreted in the light of existing evidence, which is rather inconsistent. Even when significant associations were observed, the magnitude of the correlations was still very small. Additionally, our findings are merely correlational in nature and do not provide evidence on causes of effects. Like in any type of correlational research, the interference of possible third variables cannot be completely ruled out. For instance, it is possible that working memory capacity or extraversion may explain (part of) the relation between coherence and mental health or social support (Klein and Boals, 2001; Reese et al., 2014; Vanaken and Hermans, 2020a; Van Sonderen et al., 2020).

The *second* question was whether coherence of oral and written narratives was related. We observed that only thematic coherence in oral narratives did significantly relate to written narrative coherence, as well as to the dimensions of theme and context in the written narratives. Bonferroni corrections yielded only the association of thematic oral coherence to thematic written and total written coherence significant. Thus, we only found support for the idea that the way in which individuals elaborate emotionally on autobiographical experiences and create meaning out of them could be part of a moderately stable narrative style. Possibly, the dimensions of context and chronology are more dependent on the specific event or on the modality of the narrative, and hence less reflective of the individual’s narrative style, or the ability to cognitively emotionally process and elaborate on personal experiences. This is in line with what Fivush et al. (2008) found, namely that thematic coherence seems to be a consistent individual narrative style. Nonetheless, this hypothesis would benefit from further investigation.

Despite the fact that coherence was measured for different event types (turning point for oral, high and low point for written), in different contexts (presence of experimenter vs. alone in cubicle vs. alone at home) and the fact that measures were taken 4 months apart, individuals’ level of thematic coherence is, at least to some extent, consistent in narratives of different modalities. Whilst in experimental research these differences in methods may be regarded as possible confounds, they are actually an advantage in correlational research. If associations happen to be found, and they are robust even when the circumstances are non-identical, the sturdiness of findings is only increased. For instance, it can be compared to the situation when the test–retest reliability is also interpreted as more significant and credible when measures stem from timepoints that are 4 months apart compared to taken only a week apart.

Previous research has proposed that coherence develops as a combination of a narrative style (i.e., individual’s characteristics, e.g., coping style, attachment style: Fivush et al., 2006) as well as event-specific (e.g., type of event: Banks and Salmon, 2013; emotional intensity: Rubin et al., 2011) and narration-specific aspects (e.g., context of sharing: Pasupathi, 2001). Indeed, comparing the moderate correlation between thematic oral and total written narrative coherence over a 4-month time period that was observed in the current study, *r* = 0.33, to an earlier observed correlation of two oral narrative coherence measurements over a 5-month time period, *r* = 0.48, (Vanaken et al., unpublished), suggests that there are in fact also event- and modality-specific aspects in addition to the stable narrative style that can impact coherence to some extent. This is in line with earlier work (Fivush et al., 2017; McLean et al., 2017; Adler et al., 2018; Waters et al., 2019; Pasupathi et al., 2020). Future research is nonetheless needed to investigate the extent to which each aspect adds to the equation and can explain parts of the variance in coherence, by comparing narratives across time, event, modality, etc.

Furthermore, we examined relations between oral and written narrative coherence separately for positive and negative written narratives, since negative narratives were more coherent than positive ones. This finding is actually in line with earlier research, suggesting that negative narratives call for more effort in meaning-making and hence can be more coherent than positive narratives (Fivush et al., 2002, 2003a,b, 2008; Baker-Ward et al., 2005; Vanderveren et al., 2019). Coherence in negative written narratives, was also more significantly related to the coherence in oral narratives, compared to coherence in the positive written narratives. Possibly, negative and turning point narratives are more similar than positive ones, because the former require more cognitive-emotional processing efforts compared to the latter, which tend to be shorter and sometimes lack a narrative arc (Habermas, 2019). Since this is only a *post hoc* explanation, further research is recommended.

In general, our descriptive results suggested that coherence might be higher for oral narratives than for written narratives. Since our research design was not purposely set out to test this hypothesis, multiple explanations for this finding, in terms of modality, even type, and context factors are possible. First, it could be possible that oral narratives are in general more coherent than written narratives. However, most of the evidence that compares narratives on structural variables is overall rather mixed (Tannen, 1982; Chafe and Tannen, 1987; Eaton, 2005; Özyildirim, 2009; Balon and Rimé, 2016; Drijbooms et al., 2017). Given our research design, it is hard to interpret our findings as evidence for this hypothesis.

Second, the descriptive results might equally suggest that coherence of turning point memories is higher than the coherence of high or low point memories. Evidence for this idea comes from a study of McLean et al. (2017), who looked at intra-individual variability in narrative coherence. It might be the case that turning point memories are more likely to evoke a resolution and are more practiced since they take a central position in the individual’s life.

Third, the descriptive results might be explained by contextual factors. Given that the written narratives were collected from participants at home in an online study, and the oral narratives during a lab experiment, participants might have felt more pressure to perform well on the oral narrative task, resulting in more coherent narratives. Particularly those participants that were evaluated by the experimenters whilst narrating, might have been influenced by the experimental context, more so than participants in the control condition who were seated alone in the cubicle. Nevertheless, both contexts of oral narration are still quite artificial. It is known that a real-life social context can have an influence on narrative coherence, as attentive listeners are able to co-construct a story together with the speaker during a process of social interaction (Pasupathi, 2001; Pasupathi and Billitteri, 2015). Hence, future research that compares oral and written narration in naturalistic environments is highly recommended.

Concluding, in our study, only thematic coherence of oral narratives related to outcomes of mental health and social support, like symptoms of depression, anxiety and negative social interactions. Compared to the coherence of oral narratives, the relations between written narrative coherence and outcomes of mental health and social support seemed more robust and extended. Furthermore, thematic coherence in oral narratives was significantly related to thematic and total coherence of written narratives. Relations between the coherence of oral and written narratives were stronger for written negative, than for written positive narratives. Although this manuscript offers only preliminary evidence, it is suggested that thematic coherence might be a relatively stable feature of individuals’ narrative styles that is reflected in narratives of different modalities, even when concerning different event types and collected at different moments in time.

## Data Availability Statement

The datasets presented in this study can be found in online repositories. The names of the repository/repositories and accession number(s) can be found below: Open Science Framework (10.17605/OSF.IO/9ZTR).

## Ethics Statement

The studies involving human participants were reviewed and approved by the Social and Societal Ethics Committee of the KU Leuven (G-2018 01 1067 and G-2018 10 1376). The patients/participants provided their written informed consent to participate in this study.

## Author Contributions

LV, PB, and DH: conceivement, design of the studies, and manuscript revision. LV: data collection, data analysis, and manuscript writing. PB and DH: general supervision. All authors have conceived and designed the studies and revised the manuscript.

## Conflict of Interest

The authors declare that the research was conducted in the absence of any commercial or financial relationships that could be construed as a potential conflict of interest.

## Footnotes

1 https://aspredicted.org/v62ri.pdf

2 http://aspredicted.org/blind.php?x=gh23ra

3 https://doi.org/10.17605/OSF.IO/9ZTRP
